# Extrafine beclometasone diproprionate/formoterol fumarate: a review of its effects in chronic obstructive pulmonary disease

**DOI:** 10.1038/npjpcrm.2016.30

**Published:** 2016-06-16

**Authors:** Dave Singh, Massimo Corradi, Monica Spinola, Stefano Petruzzelli, Alberto Papi

**Affiliations:** 1 Medicines Evaluation Unit, Centre for Respiratory Medicine and Allergy, The University of Manchester and University Hospital of South Manchester NHS Foundation Trust, Manchester, UK; 2 Department of Clinical and Experimental Medicine, University of Parma, Parma, Italy; 3 Chiesi Farmaceutici, Parma, Italy; 4 Department of Clinical and Experimental Medicine, University of Ferrara, Ferrara, Italy

## Abstract

A fixed-dose inhaled corticosteroid/long-acting β_2_-agonist (ICS/LABA) combination of extrafine beclometasone dipropionate and formoterol fumarate (BDP/FF) has been recently approved for use in chronic obstructive pulmonary disease (COPD). Small airway inflammation and remodelling are cardinal features of COPD; therefore, the ability of this extrafine formulation to reach the small, as well as the large, airways is likely to be therapeutically important by enabling treatment of inflammatory processes in the whole bronchial tree. The clinical development of extrafine BDP/FF has demonstrated significant benefits over extrafine FF in terms of lung function improvement and reduction of the exacerbation rate, thus supporting the beneficial effect of an ICS combined to a LABA in COPD patients. Head-to-head comparison studies versus other ICS/LABA combinations have shown that the extrafine formulation enables the clinical benefits to be achieved with a lower dose of ICS. Extrafine BDP/FF showed lung function and dyspnoea improvements comparable to other ICS/LABAs, and a significantly faster onset of action was observed when compared with a salmeterol-containing fixed-dose combination. This review summarises the clinical evidence supporting the efficacy of extrafine BDP/FF in COPD and confirming that extrafine BDP/FF achieves the type of health benefit expected from such a targeted ICS/LABA combination in COPD.

## Introduction

Chronic obstructive pulmonary disease (COPD) is a condition characterised by poorly reversible airflow limitation that is generally progressive and causes serious disability. Exacerbations and co-morbidities contribute to the overall severity in individual patients.

The Global initiative for Obstructive Lung Disease recommends that the assessment of disease severity should be multidimensional, taking into account symptoms, degree of airflow limitation and risk of exacerbations. This multidimensional severity assessment divides patients into four categories (A, B, C and D).^1^ The mainstays of drug therapy for stable COPD patients are bronchodilators, and in patients at risk of exacerbations fixed combinations of inhaled corticosteroids (ICS) and long-acting β_2_-agonist (LABA) are recommended as a first-choice treatment.^1^

Several studies have shown that a LABA combined with an ICS is more effective than the individual components alone for improving lung function and symptoms, reducing exacerbation rates and improving general health status.^2–7^ β-agonists and corticosteroids have different pharmacological targets and some synergistic interactions have been documented:

- ICS increase the β_2_-adrenoreceptor density in the airways, which can counterbalance receptor downregulation and therefore prevent the development of LABA tolerance;^8–10^
- LABAs favour the nuclear translocation of glucocorticoid receptors, therefore enhancing their mechanism of action.^11^

The clinical benefits of ICS/LABA combination treatment were demonstrated, among other studies, in the TOwards a Revolution in COPD Health (TORCH) study, a 3-year randomised trial of over 6,000 patients with moderate to severe COPD that compared inhaled fluticasone propionate (FP) and salmeterol (S) alone or in combination, with placebo.^12^ There was a 17.5% lower risk of death (absolute risk reduction of 2.6%) in the combination therapy group compared with placebo, which just missed statistical significance (95% confidence interval (CI), 0.681 to 1.002; *P*=0.052). ICS/LABA therapy reduced moderate/severe exacerbations to a greater extent compared with placebo and monocomponents. In addition, ICS/LABA treatment was associated with a slower rate of lung function deterioration compared with placebo.^13^ This benefit on the rate of lung function decline has also been observed in the SUMMIT trial, investigating the effects of fluticasone furoate/vilanterol.^14^

A fixed-dose combination of the ICS beclometasone dipropionate (BDP) and LABA formoterol fumarate (FF) has been recently approved in Europe for the treatment of COPD. This fixed-dose combination is characterised by an extrafine (i.e., mean mass aerodynamic diameter (MMAD) <2.0 μm) formulation of both active components and has a nominal dose of BDP 100 μg and FF 6 μg per actuation.^15^ The extrafine formulation enables efficient lung deposition, allowing a reduction to about half the equivalent dose of a conventional BDP aerosol and minimising systemic exposure. Small airway inflammation and narrowing are cardinal features of COPD,^16^ and the ability of this extrafine formulation to reach the small airways is therefore likely to be therapeutically important.

BDP/FF 100/6 μg is licensed for use in COPD using a pressurised metred dose inhaler (pMDI) or a dry powder inhaler (DPI). Other ICS/LABAs licensed for COPD are DPIs. Many patients prefer to use pMDIs, and the option to use this extrafine formulation using a pMDI with or without a spacer therefore offers a potentially valuable treatment option for COPD patients.^17^

This review critically evaluates the effects of extrafine BDP/FF compared with other COPD pharmacotherapies, and discusses the evidence for the potential advantages of using an extrafine combination inhaler in COPD. Two types of clinical trials involving BDP/FF were selected for inclusion in this review: (1) trials evaluating the deposition of extrafine BDP/FF in the airways of COPD patients and (2) phase 3 clinical trials in COPD patients conducted according to the regulations of the European Medical Agency (EMA) to investigate the efficacy and safety of extrafine BDP/FF in COPD patients. The focus of this article is a critical review of the strengths and limitations of three clinical trials (phase 3) of extrafine BDP/FF in COPD patients, and the relevance of the results for the treatment of COPD patients in primary care.

## Lung deposition of extrafine BDP/FF

The ability of extrafine BDP/FF to achieve central and peripheral lung deposition was investigated in an open, single-dose, parallel-group study involving 10 healthy volunteers and 8 patients with stable COPD (mean forced expiratory volume in the first second (FEV_1_) 112 and 44% of predicted, respectively).^18^ Patients inhaled four actuations of radiolabelled ^99m^Tc BDP/FF and subsequent gamma camera imaging measured activity in the entire lung and extra-thoracic region, as well as the amount of exhaled activity. Lung deposition was remarkably consistent in the two groups, and it was 34% and 33% of the nominal dose in healthy volunteers and COPD patients, respectively, with a homogeneous deposition in both large and small airways regardless of the pathophysiological condition. The amount of drug exhaled was small and ranged from 2.8 to 3.4% of the nominal dose, thus confirming that only a minimal amount of extrafine particles are exhaled.

DeBacker *et al.* used multi-slice computed tomography (CT) scans and Computational Fluid Dynamics to evaluate the effects of the extrafine BDP/FF formulation on airway geometry in COPD patients.^19^ The administration of extrafine BDP/FF led to a significant improvement in airway geometry at 4–6 h, which was greater in the lower airways compared with the upper airways. After 6 months of treatment, the hyperinflation at the lobar level at total lung capacity was significantly reduced compared with baseline values. These changes were associated with a reduction in hyperinflation measured by functional residual capacity (FRC) and improved FEV_1_. These findings indicate the efficacy of extrafine BDP/FF in improving lung physiology in the small airways.

## Study NCT00476099

The study design, primary aims and key inclusion criteria for the three clinical development studies (NCT00476099, FORWARD and FUTURE) are summarised in Table 1. NCT00476099 was a 1-year, double-blind, double-dummy, randomised, multinational, multicentre, 3-arm parallel-group trial.^20^ The primary aims were to test the non-inferiority of extrafine BDP/FF 100/6 μg versus budesonide/formoterol fumarate (BUD/FF) 200/6 μg in terms of pulmonary function (FEV_1_) and the superiority of extrafine BDP/FF versus FF in terms of exacerbation rate in COPD patients with severe airflow limitation.

The non-inferiority of extrafine BDP/FF to BUD/FF was demonstrated; the difference between the adjusted mean FEV_1_ of the extrafine BDP/FF and BUD/FF group was −0.002 l, and the lower limit of the two-sided 95% CI for the difference between groups was −0.052 l. In addition, extrafine BDP/FF led to a statistically significant improvement in FEV_1_ in comparison with FF (difference 0.051 l; 95% CI 0.001 to 0.102; *P*=0.046).

The exacerbation rate in this study was much lower (less than half) than the rates usually observed in COPD studies that have specifically studied the effects of ICS on exacerbations (Table 2), which made it difficult to draw firm conclusions on this outcome. The total number of COPD exacerbations over the 48-week treatment period were similar across all three treatment groups (~0.4 per patient per year).

At the end of the study, the mean morning pre-dose forced vital capacity (FVC) was significantly higher than baseline in the extrafine BDP/FF group (0.09 l; *P*=0.005) but not in the BUD/FF group (0.05 l; *P*=0.15) and the FF group (0.02 l; *P*=0.58), although there was no statistical difference between groups (*P*=0.39). These significant changes in FVC are likely to reflect changes in lung volumes related to improvements in distal airway function.

For quality of life, the St George's Respiratory Questionnaire showed significant improvements in all treatment groups, with no difference between treatments. The use of rescue medication at the end of the study was significantly reduced from baseline by extrafine BDP/FF (−0.27 puffs; *P*<0.001) and BUD/FF (−0.24 puffs; *P*=0.013) but not by FF (−0.04 puffs, *P*>0.05).

The mean changes in the distance covered during the six-minute walking test (6MWT) were 41.1 metres in the extrafine BDP/FF group, 35.4 metres in the BUD/FF group and 35.2 metres in the FF group (*P*<0.001 in all groups). No statistically significant differences were found in the comparisons between groups. However, only extrafine BDP/FF provided an improvement of walking ability above the threshold of 37 metres, which is thought to be a clinically significant change (the minimal clinically important difference; MCID).^21,22^ The authors suggest that the ability to walk further after treatment with BDP/FF may be linked to the improvement in air trapping.^20^

### NCT00476099: strengths

NCT00476099 was performed in patients likely to be suitable candidates for ICS/LABA treatment—i.e., severe airflow obstruction plus a history of ⩾1 exacerbation in the previous year. This was the first long-term study of extrafine BDP/FF in COPD patients, which succeeded in one of its co-primary aims, to demonstrate a similar effect on lung function compared wth BUD/FF, which is widely used in clinical practice.

### NCT00476099: Limitations

The major limitation of this study was the very low exacerbation rate observed, despite the study being conducted in severe COPD patients with a history of ⩾1 exacerbation in the previous year. This reduced the ability of the study to measure the impact of ICS/LABA compared with LABA on this outcome measure. Possible reasons for the low exacerbation rate observed include the following:

- Patients were required to be free from COPD exacerbations for 12 weeks before randomisation. This may have inadvertently biased the recruitment towards more stable patients who were less likely to exacerbate thereafter, as the best predictor of exacerbation risk is the previous history of frequent exacerbations and exacerbations tend to cluster;^23,24^
- Many patients tend to self-medicate to manage their symptom worsening.^25,26^ Many exacerbations after randomisation might not have been reported by patients, leading to under-estimation of the exacerbation frequency;
- The major risks for COPD exacerbations are community-based viral infections, such as influenza, and upper respiratory tract infections. These vary between seasons, between different years and between countries. In the period of the study conduct, the burden of respiratory tract infection/influenza over Europe was low.^27^

It should also be noted that the sample size per treatment arm was lower than in many other COPD clinical trials that have investigated the effects of inhaled treatments on exacerbations as a primary end point.

## Forward study

The primary aims of the FORWARD study were to compare extrafine BDP/FF 100/6 μg with extrafine FF 6 μg with two co-primary efficacy end points: COPD exacerbation rate over 1 year and change in pre-dose morning FEV_1_ from baseline (randomisation visit) to Week 12.^28^ The FORWARD study was designed specifically to fill the gap in evidence for the effect of extrafine BDP/FF on exacerbations by enhancing the capture of exacerbation events. Specifically, (i) patients were recruited in three ‘winter waves’ in two consecutive years across the globe (November to April for the Northern hemisphere, and April to September for the Southern Hemisphere) in order to capture the winter exacerbation peak. The validity of this approach is confirmed by the *post hoc* analysis of the TORCH study, in which a major proportion of exacerbations fell in the winter months of both Northern and Southern hemispheres;^29^ (ii) as previous exacerbation history is the most important risk factor for future exacerbations,^30^ documented evidence of an exacerbation in the last year (e.g., medical letter, hospital records) was required rather than just relying on a patient verbal report as in NCT00476099; (iii) the wash-out period free from exacerbations was limited to 6 weeks (4 weeks before screening plus 2 weeks for the run-in period), rather than 12 weeks as NCT00476099 (8 weeks before screening plus 4 weeks for the run-in period); (iv) the EXAcerbations of Chronic Pulmonary Disease Tool (EXACT)- Patient-Reported Outcome (PRO) questionnaire was used to measure symptoms. This 14-item questionnaire^31^ was recorded daily using a digital platform. The daily electronic transmission of symptom data allowed the investigators to monitor for deteriorations in health. This instrument was used also as a trigger for physicians to contact patients for evaluation of whether changes in symptoms were due to an exacerbation.

The use of tiotropium as a concomitant medication was permitted, except for a 72-h wash-out period before each clinic visit. This meant that this study was conducted in a more ‘real-life’ setting with a severe COPD population, as a large proportion (50–70%) of severe COPD patients in Western Europe use tiotropium.^32^

**Figure 1:**
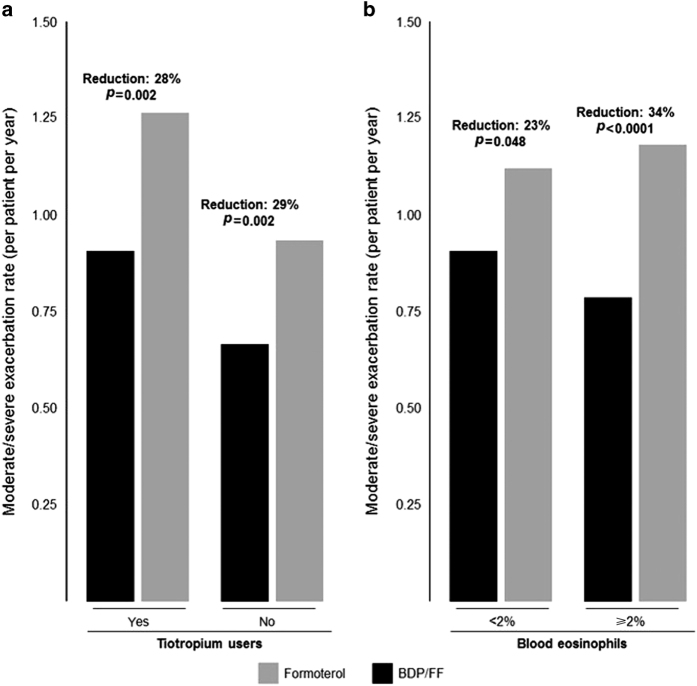
COPD exacerbations during the FORWARD study in patients stratified by different baseline characteristics. (**a**) Use of tiotropium before study entry; data from Wedzicha *et al.* ^28^ (**b**) Percentage count of baseline blood eosinophils; data on file. BDP/FF, beclometasone dipropionate/formoterol.

Extrafine BDP/FF was superior to extrafine FF in terms of the annual COPD exacerbation rate; there was a 28% reduction of moderate-to-severe exacerbations with extrafine BDP/FF compared with FF, *P*<0.001. Moreover, a subgroup analysis, stratifying patients by tiotropium use at randomisation, showed that extrafine BDP/FF was superior to FF for reduction of exacerbations both in tiotropium users and in non-tiotropium users (Figure 1a).

Extrafine BDP/FF was superior to FF also for the change in pre-dose morning FEV_1_ from baseline to Week 12, confirming the results obtained in NCT00476099. In the ITT population, the difference in the adjusted mean change between the two groups (0.069 l; *P*<0.001) in favour of extrafine BDP/FF was evident irrespective of tiotropium use. There was an improvement in the St George's Respiratory Questionnaire total score in the BDP/FF group compared with FF, with an adjusted mean difference of 2.78 units (95%: −4.51, −1.05; *P*=0.002).

### FORWARD: strengths

The sample size of FORWARD was greater than NCT00476099, thus increasing the statistical power to measure a difference between treatments on exacerbations. The key baseline characteristics of NCT00476099 and FORWARD patient populations are presented in Table 2; the populations are very similar, including the retrospectively reported exacerbation history in the previous year. However, the design of FORWARD increased the prospective capture of these events compared with NCT00476099 (~1.1 versus 0.4, respectively, for the LABA treatment arm).

The superiority of extrafine BDP/FF versus extrafine FF in terms of exacerbation reduction in tiotropium users is a novel aspect of FORWARD, as all previous ICS/LABA clinical trials have required the discontinuation of LAMA treatment. This subset analysis of tiotropium users provides useful information on the value of triple therapy (ICS+LABA+LAMA) versus dual bronchodilator therapy (LABA+LAMA) on exacerbations. There are little published data on this particular issue.

### FORWARD: limitations

FORWARD succeeded in its primary aim of demonstrating a greater effect of extrafine BDP/FF compared with FF on exacerbation reduction in severe COPD patients. The patients recruited were required to have a history of ⩾1 exacerbation in the previous year. Global initiative for Obstructive Lung Disease recommends that ICS/LABA treatments can be used in patients with ⩾2 exacerbations in the previous year. To date, no ICS/LABA clinical trials have used this inclusion criteria, so the evidence from clinical trials such as FORWARD comes from a population different from that stated by Global initiative for Obstructive Lung Disease.

FORWARD studied COPD patients with FEV_1_ <50% predicted. Some other ICS/LABA clinical trials have used an FEV_1_ cutoff up to 70% predicted,^6^ and have demonstrated an effect on exacerbations. One could assume that extrafine BDP/FF would also be effective in patients with FEV_1_ 50–70% and a history of exacerbations, but FORWARD did not specifically investigate this issue.

The FORWARD study provided the data that EU regulators required in order to grant a license for extrafine BDP/FF in COPD patients. There is debate concerning how closely the effects observed in clinical trials will predict the benefits in real-life.^33^ For example, the inclusion criteria of COPD clinical trials often exclude patients with more severe and/or symptomatic disease who are unable to withdraw inhaled medication; in FORWARD, ICS withdrawal was required during the run-in. Furthermore, patients with significant co-morbidities such as cardiovascular disease are often excluded. Clinical trials closely monitor adherence to medication, whereas in real-life this is often a significant problem. There is a need for real-life studies that overcome these issues, so that we can better understand the effects of ICS/LABA combination therapies, such as extrafine BDP/FF, in COPD patients.

### FORWARD: blood eosinophils and exacerbation rates

**Figure 2:**
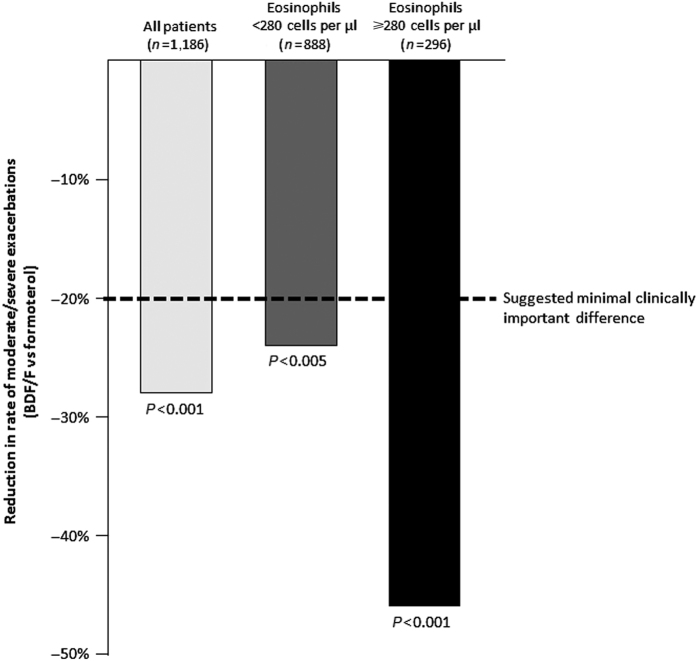
Comparison of reduction in the rate of moderate/severe exacerbations with BDP/FF versus formoterol in COPD patients. Adapted from FORWARD study: all patients (Wedzicha *et al.* ^28^) and data pooling from *post hoc* analysis on baseline blood eosinophil count (Siddiqui *et al.* ^35^). BDP/FF, beclometasone dipropionate/formoterol. 20% is the suggested minimal clinically important difference for exacerbations.^47^

Blood eosinophil count is a potential biomarker of response to ICS therapy in COPD patients.^34^ A *post hoc* analysis of the FORWARD study stratified patients into quartiles using the baseline blood eosinophil counts.^35^ The reduction in exacerbation rate with BDP/FF in comparison with FF alone ranged from 22% in patients in the lowest quartile to 46% in patients in the highest quartile. Overall, there was a comparable exacerbation rate across the ranges of eosinophil counts in patients receiving BDP/FF, whereas an increasing pattern of exacerbation rate with increasing eosinophil counts was observed in patients treated with FF alone. This suggests an increasing treatment effect size for BDP/FF in patients with higher blood eosinophil counts. A blood eosinophil count ⩾2% has been suggested as a threshold to classify patients as having ‘eosinophilic COPD’; 37% of COPD patients had eosinophil counts persistently ⩾2% over 3 years in the ECLIPSE study.^36^ Subdividing the patients in FORWARD using this threshold, Figure 1b shows a significant benefit of BDP/FF over FF in both groups, but with a larger effect size in patients with eosinophils ⩾2% (34% versus 23% reduction). Similarly, using a cutoff level of 280 cells per μl (the upper quartile in FORWARD), there is a greater benefit (46% reduction of exacerbations) in patients with higher eosinophils (>280 cells per μl), but still a significant effect (24%) in patients with eosinophil counts below this threshold (Figure 2). This was a *post hoc* analysis, and thus it was not statistically powered to define the effect sizes in the quartiles. Nevertheless, the ICS effect appears to increase with higher eosinophil counts, possibly because of exacerbations that are not effectively treated with bronchodilators. These results from a *post hoc* analysis should be confirmed in prospective clinical trials, to confirm the observation and provide further guidance on the blood eosinophil cutoff level(s) that could be used in clinical practice.

## Future study

This was a 12-week multicentre, multinational, randomised, double-blind, double-dummy, 2-arm parallel-group design comparing extrafine BDP/FF 100/6 μg (*n*=211) with FP/S (Seretide, Accuhaler GlaxoSmithKline, Middlesex, UK, 500/50 μg; *n*=208) in patients with moderate to severe COPD.^37^ The primary objectives of the study were to demonstrate the superiority of extrafine BDP/FF versus FP/S, in terms of pulmonary function (FEV_1_ standardised area under the curve between time 0 and 30 min (AUC_0-30min_)) after drug inhalation on the morning of Day 1, and the equivalence between treatments in terms of the Transition Dyspnoea Index (TDI) score at week 12. St George's Respiratory Questionnaire was also assessed at baseline and at week 12.

The mean (±s.d.) FEV_1_ AUC_0-30min_ was greater in the extrafine BDP/FF group (0.17±0.13 l) compared with the FP/S group (0.09±0.10 l) on day 1; the adjusted mean difference between groups was 0.073 l (95% CI: 0.050 to 0.095, (*P*<0.001). This difference between treatments was also demonstrated after dose at week 12. In patients with FEV_1_% predicted<50%, similar results were observed.

The mean (±s.d.) TDI score was 1.47±2.64 with extrafine BDP/FF and 1.31±2.86 with FP/S; the adjusted mean difference between groups was 0.165, with the 95% CI for the difference (−0.387 to 0.718) lying entirely within the pre-defined±1 equivalence margins (*P*=0.56 between groups). A TDI score ⩾1 (the MCID^21^) was observed in 93 patients (44.1%) in the BDP/FF group and in 89 (43.0%) in the FP/S group (*P*=0.92 between groups).

Secondary efficacy end point measurements showed no difference between treatments, including the pre-dose FEV_1_. Both treatments improved the quality of life (St George's Respiratory Questionnaire), but only extrafine BDP/FF provided an improvement greater than the 4-unit MCID.

### FUTURE: strengths

This is the first head-to-head study comparing extrafine BDP/FF with one of the most commonly used drugs for COPD patients (FP/S), evaluating different ICS dosages in fixed combination therapies. ICS can cause side effects such as osteoporosis and pneumonia.^38^ FUTURE demonstrated that a lower ICS dose in extrafine BDP/FF compared with high-dose FP/S resulted in equivalent improvements in dyspnoea and trough FEV_1_. This may be important when considering the potential of ICS to cause long-term side effects.

A potential advantage of extrafine BDP/FF over FP/S is the significantly greater effect immediately after dosing in the morning that is present at the first dose and also persists during long-term treatment. This is likely to be due to the well-known faster onset of action of formoterol compared with salmeterol.^39^ Early-morning symptoms are common for many COPD patients,^40^ and an ICS/LABA with a faster onset of bronchodilation may be very useful for such patients.

### FUTURE: limitations

FUTURE provided a comparison of extrafine BDP/FF and FP/S over 12 weeks. The duration of the study was short for measuring exacerbations, and the population recruited was not enriched for a history of exacerbations. The similar effect of the extrafine BDP/FF and FP/S on other outcome measurements suggests that exacerbation reduction would also be similar, but this conclusion cannot definitively be drawn.

## Safety

NCT00476099 and the FORWARD study were long-term studies that provided relevant information on adverse events (AEs). In the NCT00476099 study, the incidence of AEs were similar between treatments: 42.8% of patients in the extrafine BDP/FF group, 40.9% in the BUD/FF group and 44.1% in the FF group, with AEs leading to study discontinuation reported in only 9 (3.8%), 6 (2.5%) and 5 (2.1%) patients, respectively. A similar pattern in AEs leading to discontinuation was observed in FORWARD: 26 (4.3%) and 28 (4.7%) for BDP/FF and FF, respectively.

Pneumonia has been identified as a risk for COPD patients treated with ICS/LABA combinations.^41^ The overall incidence of such events has generally been reported as low (<10%; Table 3) and should be interpreted in the overall context of such risks against the benefits of reducing exacerbations and hospitalisation for life-threatening events. It should also be noted that the increase risk of occurrence of pneumonia with ICS-LABAs compared with the corresponding monotherapy is not associated with increased mortality.^42^ In the NCT00476099 study, pneumonia was reported in only five patients (2.1%) in the extrafine BDP/FF group, seven (2.9%) patients in the BUD/FF group and in one (0.4%) patient in the FF group. In the FORWARD study, pneumonia was reported by 23 patients (3.8%) in the extrafine BDP/FF group and 11 patients (1.8%) in the FF group. Overall, the rate of pneumonia observed in the two long-term BDP/FF studies was very low; it is generally in line with those of a meta-analysis on long-term use of ICS and risk of pneumonia in COPD.^43^

## NCT00476099 and FORWARD; comparison with other ICS/LABA studies

For COPD patients with a history of exacerbations, international guidelines recommend the use of an ICS/LABA combination based on evidence showing significant improvement of lung function, symptoms and health status, and significant reduction of exacerbations.^1^ The clinical trial programme for extrafine BDP/FF has shown that this combination has a greater effect than the FF component alone on lung function in NCT00476099 and FORWARD. The improvements in pre-dose FEV_1_ at the end of NCT00476099 and FUTURE (0.077 and 0.07 l, respectively) were similar to the effects reported in clinical trials of other ICS/LABA combinations, as shown in Table 4;^2–7,20,28,44^ for most studies, the effect was between 50 and 90 ml. BDP/FF caused a 28% reduction of exacerbations compared with FF in FORWARD; again, this is similar to the effect sizes reported on other ICS/LABA studies, which generally varies between 25 and 30%^3–7,12,28,44–46^ (listed in Table 5). There is no direct comparison of the effect of BDP/FF with other ICS/LABA combinations on exacerbation rate reduction, but the indirect comparison with the results in Table 5 indicates that BDP/FF has similar effects to the ICS/LABA combinations FP/S and BUD/FF that are commonly used in clinical practice.

## Clinical trial data for extrafine BDP/FF; relevance to primary care

Clinical trials of ICS/LABA combinations have generally been conducted using criteria that exclude many patients who would receive these medicines in a primary-care setting in real-life, as already discussed. There is a need for real-life studies that evaluate the effectiveness of ICS/LABA combinations, including extrafine BDP/FF. Real-life studies also allow the impact of patient compliance of treatment effectiveness to be assessed. In the absence of such studies, we are left with the information from randomised controlled trials to make treatment decisions.

ICS/LABA combinations are frequently prescribed in both primary and secondary care. FORWARD recruited from both primary and secondary care clinics, and thus the results support the use of extrafine BDP/FF in both these clinical settings.

ICS/LABA combinations including FP/S and BUD/FF have historically been widely used to treat COPD patients in primary care. The direct comparison of extrafine BDP/FF with FP/S in the FUTURE study, and the indirect comparisons presented in Tables 4 and 5, indicate similar effects of extrafine BDP/FF compared with other ICS/LABA combination inhalers. In this situation, the decision of a health-care professional regarding which inhaler to use will be influenced by factors other than efficacy, such as cost and inhaler device preference.

BDP/FF is licensed for use in COPD patients with both a pMDI and a DPI. Other ICS/LABA combinations are licensed for COPD using DPI devices. The pMDI option that extrafine BDP/FF provides is likely to be useful practically for many COPD patients in primary care.^17^

## Conclusions

Extrafine BDP/FF is the only ICS/LABA approved for use in COPD patients as both a pMDI and a DPI. This allows health-care professionals to select the most appropriate inhaler based on specific patient needs. The extrafine formulation enables drug delivery from both inhalers to both the large and small airways, and allows the clinical benefits to be achieved with a lower ICS dose compared with larger-particle ICS/LABA combinations. The clinical studies performed show a benefit of extrafine BDP/FF over FF in terms of lung function and the risk of exacerbations that is comparable to the effect sizes observed for other ICS/LABA combinations. Overall, the clinical development of extrafine BDP/FF demonstrates that this extrafine formulation achieves the type of health benefits expected from such a targeted ICS/LABA combination.

## Figures and Tables

**Table 1:** Main characteristics of COPD studies with BDP/FF

*Name of the study*	*Treatments*	*Study duration*	*Rand. patients*	*Primary end points*	*Key inclusion criteria*
NCT00476099^20^	BDP/FF (200/12 μg) BUD/FF (400/12 μg) FF (12 μg) BID	48 weeks	718	Change from baseline to 48 weeks in pre-dose FEV_1_ and mean rate of COPD exacerbations.	Age ⩾40 years ⩾20 pack-years PB FEV_1_ 30%÷50% of pred. and FEV_1_/FVC<0.7 One severe exacerbation in the previous year Change in FEV_1_ <12% of pred. after salbutamol Free from COPD exacerbations for 12 weeks before randomisation
FORWARD^28^	BDP/FF (200/12 μg) FF (12 μg) BID	48 weeks	1199	Change from baseline to 12 weeks in pre-dose morning FEV_1_ and mean rate of COPD exacerbations	Age >40 years ⩾10 pack-years PB FEV_1_ 30%÷50% of pred. and FEV_1_/FVC<0.7 Documented history of at least one exacerbation in the previous year
FUTURE^37^	BDP/FF (200/12 μg) FP/S (500/50 μg) BID	12 weeks	419	TDI score at week 12, and AUC_0-30min_ at randomisation	Age ⩾ 40 years ⩾10 pack-years PB FEV_1_ 30%÷60% of pred. and FEV_1_/FVC<0.7 BDI⩽10 An increase in FEV_1_ ⩾ 5% from baseline following administration of salbutamol

Abbreviations: AUC, area under the curve; BDI, baseline dyspnoea index; BID, bis in die; BDP, beclometasone; BUD, budesonide; FF, formoterol fumarate; FP, fluticasone; FVC, forced vital capacity; PB, postbronchodilator; pred., predicted; S, salmeterol, TDI, transitional dyspnoea index.

**Table 2:** Demographic and other baseline characteristics of NCT00476099 and FORWARD studies

	*NCT00476099*			*FORWARD*	
	*BDP/FF (*N*=232)*	*BUD/FF (*N*=238)*	*FF (*N*=233)*	*BDP/FF (*N*=595)*	*FF (*N*=591)*
Male, *n* (%)	184 (79.3)	194 (81.5)	189 (81.1)	408 (68.6)	410 (69.4)
Female, *n* (%)	48 (20.7)	44 (18.5)	44 (18.9)	187 (31.4)	181 (30.6)
Age, years, mean (s.d.)	63.0 (9.0)	64.1 (9.1)	63.7 (8.8)	64.6 (8.6)	63.9 (8.6)
BMI, kg/m^2^, mean (s.d.)	26.8 (5.3)	26.6 (5.0)	26.7 (4.7)	26.5 (5.4)	26.5 (5.3)
FEV_1_, *L*, mean (s.d.)	1.22 (0.3)	1.23 (0.3)	1.22 (0.3)	1.15 (0.3)	1.16 (0.3)
FEV_1_, % pred. mean (s.d.)	41.9 (5.8)	42.3 (6.0)	42.5 (5.9)	41.86 (6.0)	41.61 (6.0)
FVC, *L*, mean (s.d.)	2.46 (0.6)	2.47 (0.6)	2.46 (0.6)	2.46 (0.7)	2.52 (0.7)
FEV_1_/FVC, mean (s.d.)	0.51 (0.1)	0.51 (0.1)	0.51 (0.1)	0.48 (0.1)	0.48 (0.1)
Disease duration, years, mean (s.d.)	9.4 (7.0)	9.9 (7.8)	9.8 (6.7)	7.9 (5.9)	7.5 (5.7)
Exacerbations/patient, mean (s.d.)	1.7 (1.0)	1.7 (1.0)	1.8 (1.0)	1.5 (0.9)	1.4 (0.9)
Current smokers, *n* (%)	90 (38.8)	86 (36.1)	87 (37.3)	231 (38.8)	237 (40.1)
Ex-smokers, *n* (%)	142 (61.2)	152 (63.9)	146 (62.7)	364 (61.2)	354 (59.9)
Pack-years, mean (s.d.)	37.3 (14.1)	37.8 (14.6)	39.7 (19.1)	43.1 (23.5)	42.7 (22.9)
Tiotropium users at randomisation, *n* (%)	0 (0.0)	0 (0.0)	0 (0.0)	318 (53.4)	298 (50.4)
SGRQ total, mean (s.d.)	51.0 (15.4)	49.7 (15.8)	50.6 (16.2)	47.3 (17.9)	48.0 (17.2)

Abbreviations: BMI, body mass index; FVC, forced vital capacity; SGRQ, St George's Respiratory Questionnaire.

**Table 3:** Percentage of patients with pneumonia in recent studies investigating ICS/LABA combinations versus LABA monotherapy in COPD patients

*Study reference*	*Drugs and total daily dose (μg)*	*Study duration*	*% of patients with pneumonia*	
			*ICS/LABA*	*LABA*
Calverley *et al.* ^12^	FP/S 1000/100 versus S 100	3 years	19.6	13.3
Kardos *et al.* ^45^	FP/S 1000/100 versus S 100	44 weeks	4.5	1.4
Calverley *et al.* ^3^	BUD/FF 640/18 versus FF 18	12 months	3.1	2.7
Ferguson *et al.* ^4^	FP/S 500/100 versus S 100	12 months	7.0	4.0
Rennard *et al.* ^7^	BUD/FF 320/9 versus FF 9	12 months	3.0	3.4
Rennard *et al.* ^7^	BUD/FF 160/9 versus FF 9	12 months	3.0	3.4
Anzueto *et al.* ^46^	FP/S 500/100 versus S 100	52 weeks	6.6	2.5
Carlverley *et al.* ^20^	BDP/FF 400/24 versus FF 24	48 weeks	2.1	0.4
Carlverley *et al.* ^20^	BUD/FF 800/24 versus FF 24	48 weeks	2.9	0.4
Sharafkhaneh *et al.* ^44^	BUD/FF 320/9 versus FF 9	12 months	6.4	2.7
Sharafkhaneh *et al.* ^44^	BUD/FF 160/9 versus FF 9	12 months	4.7	2.7
Dransfield *et al.* ^6^	FF/Vil 100/25 versus Vil 25	52 weeks	6.3	3.3
Wedzicha *et al.* ^28^	BDP/FF 400/24 versus FF 24	48 weeks	3.8	1.8

Studies >6 months duration were included.

Abbreviations: COPD, chronic obstructive pulmonary disease; ICS/LABA, inhaled corticosteroid/long-acting β_2_-agonist.

**Table 4:** Pre-dose FEV_1_ improvements in studies of ICS/LABA combinations in COPD

*Clinical studies*	*Pre-dose FEV*_*1*_ *at baseline*^a^ *(ml)*	*Pre-dose FEV*_*1*_ *versus baseline*^b^ *(ml)*	*Pre-dose FEV*_*1*_ *versus LABA*^b^ *(ml)*	*Timepoint considered for FEV*_*1*_ *comparison*
Calverley^2^^,^^c^	1,266	120	73	52 weeks
Ferguson^4^^,^^c^	950	102	74	52 weeks
Szafranski^5^^,^^d^	960	80	80	12 months
Calverley^3^^,^^d^	980	N/A	50	12 months
Wedzicha^28^^,^^e^	1,052	70	65	48 weeks
Calverley^20^^,^^e^	1,140	77	50	48 weeks
Rennard^7^^,^^d^	1,020	120	90	12 months
Sharafkhaneh^44^^,^^d^	1,000	70	30	12 months
Dransfield^6^^,^^f^	N/A	N/A	40	52 weeks

Studies >6 months duration were included. Studies with pre-dose FEV_1_ presented were included.

Abbreviations: LABA, long-acting β_2_-agonist; N/A, not available.

a FEV_1_ before first dose (baseline).

b Change in FEV_1_ caused by ICS/LABA.

c Studies conducted with: fluticasone propionate/salmeterol.

d Budesonide/formoterol fumarate.

e Beclometasone dipropionate/formoterol fumarate.

f Fluticasone furoate/vilanterol.

**Table 5:** COPD exacerbation annual rate in recent studies of ICS/LABA combinations

*Study reference*	*Drugs and total daily dose (μg)*	*Study duration*	*Annual rate of moderate/severe exacerbations*		
			*ICS/LABA*	*LABA*	*Reduction ICS/LABA versus LABA (%)*
Calverley *et al.* ^3^	BUD/FF 800/24 versus FF 24	12 months	1.38	1.85	25.5^a^
Calverley *et al.* ^12^	FP/S 1000/100 versus S 100	3 years	0.85	0.97	12^a^
Szafranski *et al.* ^5^	BUD/FF 800/24 versus FF 24	12 months	1.42	1.84	23^a^
Kardos *et al.* ^45^	FP/S 1000/100 versus S 100	44 weeks	0.92	1.4	35^a^
Ferguson *et al.* ^4^	FP/S 500/100 versus S 100	12 months	1.06	1.53	30.5^a^
Rennard *et al.* ^7^	BUD/FF 400/12 versus FF 12	12 months	n/a	n/a	25^a^
Rennard *et al.* ^7^	BUD/FF 200/12 versus FF 12	12 months	n/a	n/a	29^a^
Anzueto *et al.* ^46^	FP/S 500/100 versus S 100	52 weeks	1.10	1.59	30.4^a^
Sharafkhaneh *et al.* ^44^	BUD/FF 400/12 versus FF 12	12 months	0.70	1.07	34.6^a^
Sharafkhaneh *et al.* ^44^	BUD/FF 200/12 versus FF 12	12 months	0.79	1.07	25.9^a^
Dransfield *et al.* ^6^	FF/Vil 100/25 versus Vil 25	52 weeks	0.81	1.11	30^b^
Wedzicha *et al.* ^28^	BDP/FF 400/24 versus FF 24	48 weeks	0.80	1.12	28.1^a^

Studies >6 months were included.

NCT00476099 excluded from this summary, because of a low exacerbation rate.^20^

Abbreviations: BDP/FF, beclometasone dipropionate/formoterol fumarate; BUD/FF, budesonide/formoterol fumarate; FP/S, fluticasone propionate/salmeterol; FF/Vil, fluticasone furoate/vilanterol.

a *P* value ICS/LABA versus LABA alone <0.05.

b *P* value not available.
